# Increased adiposity and impaired sleep are associated with severity of greater trochanteric pain syndrome: a cross-sectional study

**DOI:** 10.3389/fmed.2025.1718267

**Published:** 2025-12-03

**Authors:** Marco Alessandro Minetto, Chiara Busso, Marta Andrighetti, Elisabetta Quilico, John A. Shepherd, Steven B. Heymsfield, Angelo Pietrobelli

**Affiliations:** 1 Division of Physical Medicine and Rehabilitation, Department of Surgical Sciences, University of Turin, Turin, Italy; 2 Department of Epidemiology, University of Hawaii Cancer Center, Honolulu, HI, United States; 3 Pennington Biomedical Research Center, Baton Rouge, LA, United States; 4 Paediatric Unit, Department of Surgical Sciences, Dentistry, Gynecology and Pediatrics, University of Verona, Verona, Italy

**Keywords:** body composition, digital anthropometry, obesity, pain drawing, physical performance, tendinopathy

## Abstract

**Background:**

To investigate the feasibility and clinical validity of a digital anthropometric approach for body size and composition assessment in patients with greater trochanteric pain syndrome and to examine physical, sleep, and pain characteristics in different subgroups of patients.

**Methods:**

A convenience sample of 62 female patients was recruited. Administration of questionnaires, pain drawing analysis, evaluation of physical performance, body size and composition assessments were performed.

**Results:**

Pain intensity was significantly higher during evening (median value of 5) compared to both morning and afternoon (median values of 4). The median values of the Pittsburgh Sleep Quality Index (PSQI) global score and of sleep duration were 9.0 and 6.0 h, respectively. The median values of body fat percentage and fat mass index were 35.2% and 9.4 kg/m^2^. Significant differences were observed between different subgroups of patients (low vs. high severity of tendinopathy-related disability) for the following variables: PSQI global score and proportion of patients with poor sleep quality (PSQI score > 5), body mass index, waist circumference, body roundness index, hip circumference, and fat mass index.

**Conclusion:**

Tendinopathic patients presented an impairment of sleep quality and quantity and an increased central adiposity that can be documented through clinimetric and body composition assessments.

## 1 Introduction

Greater trochanteric pain syndrome, also known as gluteal tendinopathy, presents with pain and tenderness over the greater trochanter that interfere with physical function and impact on the quality of life (1–3). Clinical risk factors for the development of this tendinopathy include older age, female gender, back pain, poor hip abduction function, altered gait parameters, and psychological distress (4, 5). The observation that increased trochanteric width and gynoid adiposity were associated with this tendinopathy suggested that the mechanical overload (i.e., local compression of the tendon against the greater trochanter) may represent an additional risk factor for this disorder (6). However, increased adiposity has been proposed as an under-recognized risk factor not only for greater trochanteric pain syndrome, but for all tendinopathies (7–9) because of possible systemic mechanisms, such as increased cytokine levels and chronic low-grade inflammation, influencing tendon structure and predisposing to tendon pathology (10). However, no (6) or minimal inter-group differences (4) in body mass index (BMI), which is a globally applied phenotypic descriptor of adiposity, were found in cross-sectional studies comparing healthy and tendinopathic subjects (6) or subgroups of patients with different severity of greater trochanteric pain syndrome (4). Similarly, no differences in BMI were observed between subjects with asymptomatic Achilles tendinopathy and healthy controls, although the former group showed increased (central) adiposity compared to the latter group (9). Therefore, it has been suggested that in addition to BMI, measures of adiposity and its distribution should be reported in musculoskeletal studies (6). Recent technological advances have made available for physicians new tools such as optical body scanners that capture a three-dimensional image of the body and provide useful biomarkers of body size, shape, and composition (11, 12) that enable to move beyond BMI to precisely characterize the health status and the metabolic profile (13). However, no previous study, to our knowledge, was performed in patients with greater trochanteric pain syndrome to quantify their body composition changes through digital anthropometry.

Recent studies also showed that sleep problems can be considered key pathophysiological factors in different non-malignant musculoskeletal painful conditions (14, 15). Consistently, patients with sleep problems reported greater use of sleep and pain medications than those sleeping normally (16). Although tendinopathic patients frequently report that greater trochanteric pain may also interfere with sleep (3, 17), no previous study, to our knowledge, was performed in patients with greater trochanteric pain syndrome to investigate their sleep characteristics. Therefore, the primary aim of this study was to investigate the feasibility and clinical validity of a digital anthropometric approach for body size and composition assessment in patients with greater trochanteric pain syndrome. The secondary study aim was to examine physical, sleep, and pain characteristics in subgroups of patients presenting with varying levels of perceived disability, in order to identify important attributes or problems that are different for different clinical phenotypes.

## 2 Materials and methods

### 2.1 Participants and protocol

A convenience sample of 62 female patients [median age of 59.0 (1st – 3rd quartile: 51.0 – 68.0) years] was recruited. Inclusion criteria were the presence of chronic lateral hip pain exacerbated by activity and by lying on the affected side (4, 5), and clinical diagnosis of gluteal tendinopathy performed by a medical doctor and confirmed by the ultrasound evidence of one or more of the following findings: decreased and heterogeneous echogenicity of the gluteus minimus and/or medius tendons, tendon thickening, calcification at the tendon attachment with the greater trochanter, cortical irregularities deep to the gluteal insertions, bursal fluid collection (18). Exclusion criteria were: hip osteoarthritis, neurological or systemic inflammatory diseases, lumbar spine nerve root signs, history of lumbar spine or ipsilateral hip joint surgery (19), presence of cognitive disturbances that could influence completion of self-administration questionnaires and pain drawings.

Clinical evaluation, administration of outcome and pain questionnaires, anthropometric assessment, and physical performance assessment were performed in a single experimental session.

All patients gave their written consent after receiving a detailed explanation of the protocol. The study conformed to the guidelines of the Declaration of Helsinki and was approved by the local ethics committee (protocol n. 0065654).

### 2.2 Clinimetric assessments of patient characteristics

The following patient characteristics were investigated according to the items of the ICON PART-T consensus (20, 21): pain phenotype measures (duration, intensity, interference, extension and location, number of pain areas), analgesic medication use, level of perceived disability, level of physical activity, sleep quality, and physical function.

Pain duration was queried using a 6-interval scale (less than 1 month, 1–3 months, 3–6 months, 6–12 months, 1–2 years, more than 2 years).

Pain intensity and interference assessment was performed with the Italian version of the Brief Pain Inventory (22) that quantifies pain intensity with 4 items (worst, least, average, and right now), activity pain interference with 3 items (general activity, walking, work), and affective pain interference with 4 items (mood, relation with other people, sleep, enjoyment of life). All items are scored using an 11-point numerical rating scale (with 0 corresponding to “no pain”/“no interference” and 10 corresponding to “the worst imaginable pain”/“maximal interference”). For this study, we used the mean score of the 4 items for pain intensity, of the 3 items for activity pain interference, and of the 4 items for affective pain interference.

The pain intensity time course was evaluated through a pain diary: each patient was instructed to rate the pain intensity (between 0 and 10) thrice daily (at 8:00, 16:00, 23:00) for seven consecutive days.

Pain extension and location were assessed through a digital device featuring body charts templates and automated analysis of pain drawing, as previously described (23, 24). Lateral body chart view was adopted: patients were instructed to color every part of the affected lower limb where they perceived pain, regardless of pain type and severity (the most symptomatic side was considered in patients presenting with bilateral pain). The researchers provided the following standardized instruction: “Please draw where you felt your usual pain during the last week on this body chart and try to be as precise as possible”. Pain extension was quantified as the total number of pixels colored within the body chart perimeter, while pain location was assessed through a pain frequency map. Briefly, the pain drawings of all patients were superimposed to obtain a map with different colors indicating different percentages of patients reporting pain in a specific area (the lower limb of the right side was arbitrarily selected for all patients to represent the pain frequency map - see section “3. Results”).

Patients were asked to indicate their preferred analgesic treatment (used as needed) and to rate its pain-relieving effect on a 4-point Likert scale with the following response options: 0, no effect; 1, mild effect; 2, moderate effect; 3, strong effect.

Patients were also asked to complete the following self-administration questionnaires in a standardized order: Italian version (24) of the Victorian Institute of Sports Assessment for Gluteal Tendinopathy (VISA-G) questionnaire (19), polysymptomatic distress (PSD) scale (25), Italian version (26) of the Pittsburgh Sleep Quality Index (PSQI) questionnaire (27), and Italian version (28) of the short International Physical Activity Questionnaire (IPAQ) (29).

The VISA-G questionnaire is the preferred available option to capture the disability associated with gluteal tendinopathy (30). It consists of eight questions that measure the domains of pain, function in daily living, and sports activities. The cut-points previously reported by Plinsinga et al. (4) were adopted to distinguish between different subgroups: VISA-G score ≤50% for severe disability, VISA-G score between 51% and 67% for moderate disability, and VISA-G score ≥68% for mild disability.

The PSD scale consists in the evaluation of the following two components: (i) widespread pain index (WPI) that is a 0–19 count of the following body regions: back of the neck, upper back, lower back, chest, abdomen, left and right side of the jaw, shoulder girdle, upper arm, lower arm, hip (trochanter included), upper leg (knee included), lower leg (ankle and foot included), (ii) symptom severity scale (SSS) that is a 0–12 measure of symptom severity including fatigue, waking unrefreshed, and cognitive problems. The PSD score can be obtained by summing the WPI and SSS scores and can be used to identify patients with fibromyalgia. A patient satisfies the fibromyalgia diagnostic criteria if: (i) the pain is generalized (i.e., present in at least 4 of the following 5 regions: left upper arm, right upper arm, axial region with the exclusion of chest and abdomen, left lower limb, right lower limb), and (ii) WPI score is ≥7 and the SSS score is ≥5 (or WPI is ≥4 and SSS score is ≥9) (25).

The PSQI questionnaire contains 19-self rated questions that produce a global sleep quality score (with a range between 0 points, indicating no difficulty, and 21 points, indicating severe difficulties in all areas) and the following seven component scores (each of which has a range of 0–3 points, with higher scores indicating greater difficulty): sleep quality, sleep latency, sleep duration, habitual sleep efficiency, sleep disturbance, use of sleeping medication, and daytime dysfunction. Sleep duration is assessed through the following open-ended question: “During the past month, how many hours of actual sleep did you get at night?”. Participants who had reported sleeping for <7 h were classified as short sleepers. This cut-off was chosen based on the Joint Consensus Statement of the American Academy of Sleep Medicine and Sleep Research Society, which recommends 7 h of sleep as the lowest sleep duration to be appropriate for optimal health in adults (31). The overall sleep quality was defined based on a cut-off of 5 on the global PSQI score to distinguish between good sleepers (score ≤ 5) and poor sleepers (score > 5) (26, 27).

The IPAQ short comprises seven items investigating different physical activity intensities (vigorous or moderate), the time spent walking and sitting (as a proxy for sedentary behavior) during the last 7 days. Based on IPAQ results, three levels of physical activity were proposed in a categorical score: (1) low physical activity level (sedentary subjects): IPAQ score below 600 MET × min/week; (2) moderate physical activity level (moderately active subjects): IPAQ score equal to or above 600 MET × min/week and below 3000 MET × min/week; (3) high physical activity level (active subjects): IPAQ score of at least 3000 MET × min/week (29).

### 2.3 Anthropometric and body composition assessments

Body weight and height were assessed with each patient in undergarments and barefoot. Body weight and height were measured (to the nearest 0.1 kg and 0.5 cm, respectively) using a standard scale with a stadiometer (model Seca 799, Seca GmbH & Co., KG, Hamburg, Germany).

Optical images were taken with ProScanner device (version 5.0, Fit3D, Sacramento, CA, USA), using a standardized protocol, as previously described (32). Briefly, each patient was asked to stand upright in a standardized A-pose (with shoulder relaxed and arms positioned straight and abducted from the torso) while grasping the telescoping handles. A full body scan takes ∼ 45 s during which light-coding depth sensors capture the tri-dimensional shape as the platform rotates once around. The acquisition of the tri-dimensional shape was performed in duplicate to obtain two avatars for each patient. Each avatar consists in a mesh connected by triangles with approximately 300,000 vertices and 600,00 faces to represent body shape. The raw mesh files were digitally registered and reposed using Meshcapade (Meshcapade GmbH, Tübingen, Germany) (33, 34) to obtain the average avatar for the whole sample of patients (see section “3. Results”). Along with the mesh, the Fit3D dashboard provides also for each avatar a large number of anthropometric measurements (i.e., whole-body and segmental circumferences, lengths, surface areas, volumes) and body composition estimates. The following body size, shape, and composition variables obtained by proprietary algorithms were considered (data obtained for the two avatars were averaged): body mass index, waist circumference, hip circumference, waist-to-hip ratio, body fat percentage, fat mass index and fat free mass index (i.e., fat mass and fat free mass scaled to height squared, respectively). Moreover, appendicular lean mass index (i.e., sum of the soft lean tissue in the arms and legs scaled to height squared) and body roundness index (i.e., an index combining waist circumference and height that can range between 1 and 20, where 1 represents more narrow body types and 20 represents more round body types: the healthy range is about 4–5) were obtained according to previously published equations (Supplementary Table 1) (35, 36). In a female population, waist circumference ≥88 cm and waist-to-hip ratio >0.85 can be considered indicative of increased waist circumference and central obesity, respectively, (37) while fat mass index >9 kg/m^2^ indicates excess fat (38) and appendicular lean mass index <5.45 kg/m^2^ is a proxy for low muscle mass (39).

### 2.4 Physical performance assessment

The 5-repetition-sit-to-stand test was performed to evaluate the lower limb strength: patients were asked to stand and sit from an armless chair (of standard height: 48 cm) 5 times (with their arms crossed over their chest) and the time to complete the test was recorded. The test was repeated twice and the best of the two performances was considered. Poor performance was identified according to previously published normative values for healthy females (40): test time >9.3 s in patients aged ≤60 years and >13.4 s in patients aged >60 years.

### 2.5 Statistical analysis

**FIGURE 1:**
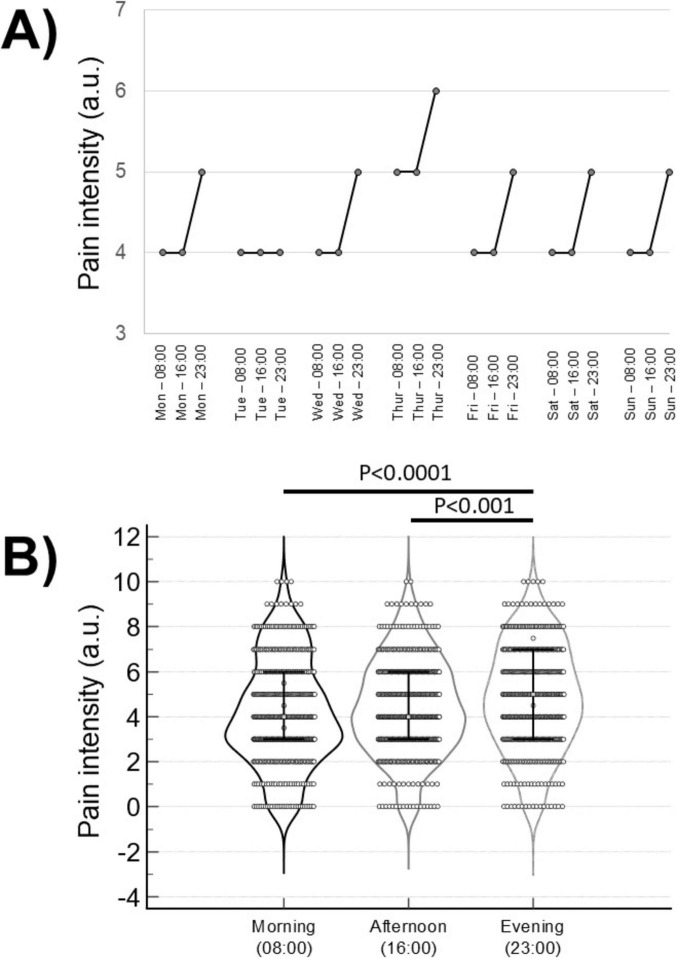
**(A)** Time course (over a 7-d period) of the median pain intensity, assessed using a 0–10 numerical rating scale, self-reported thrice (at 8:00, 16:00, and 23:00) daily. **(B)** Violin plots of the pain intensity self-reported thrice (at 8:00, 16:00, and 23:00) daily (data of different days were pooled). Error bars indicate the median values and the interquartile ranges.

The Shapiro–Wilk test for normal distribution of the data failed, therefore non-parametric statistical tests were used. Friedman’s ANOVA followed by Dunn’s multiple comparisons test were adopted to analyse the pain intensity time course (i.e., the differences among the pain intensity values reported by the patients at different time points – see Figure 1B).

K-means algorithm cluster analysis was adopted to classify patients into the low and high disability subgroups on the basis of the VISA-G scores. The Fisher’s exact test and the Mann-Whitney U test were adopted for comparisons between the two subgroups.

Data were expressed as median and 1st – 3rd quartiles and were represented with violin plots (in Figure 1B) showing the probability density functions of the data sets. The threshold for statistical significance was set at *P* = 0.05. Statistical tests were performed using the SPSS v. 20.0 (SPSS Inc., Chicago, IL, USA) software package.

## 3 Results

Gluteal tendinopathy affected the right side in 18 patients (59 patients were right side dominant), the left side in 26 patients, while 18 patients reported bilateral pain.

Three-fourths (47 of 62) of the patients used analgesic drugs as needed with variable pain relief (no effect in 2 patients, mild in 11 patients, moderate in 20 patients, and strong in 14 patients).

Descriptive data of clinimetric scores, physical function, and pain phenotype variables are reported in Table 1: pain-related disability was from moderate to severe in most of the patients (28 of 62 patients showed VISA-G score between 51% and 67%, while 19 of 62 patients showed VISA-G score ≤50%). Most of the patients were sedentary (19 patients) or moderately active (29 patients) and most of them showed a poor physical function (39 of 62 patients).

**TABLE 1:** Median (1st–3rd quartile) values of the results of the clinimetric and physical performance assessments obtained in the whole group of 62 patients.

Variable	Value
VISA-G score (0–100)	60.0 (48.3 – 67.0)
**IPAQ score**	
(MET × min/week)	1284.0 (463.0–2725.5)
**5-rep-sit-to-stand test (s)**	
• ≤60 years (*n* = 34 patients)	11.8 (10.0–12.9)
• >60 years (*n* = 28 patients)	11.7 (10.4–13.7)
Global PSQI score (a.u.)	9.0 (6.0 – 12.0)
**Component scores:**	
• Sleep quality (a.u.)	(1.0 – 2.0)
• Sleep latency (a.u.)	(0.3 – 2.0)
• Sleep duration (a.u.)	(1.0 – 2.0)
• Habitual sleep efficiency (a.u.)	(0.0 – 2.0)
• Sleep disturbance (a.u.)	2.0 (1.0 – 2.0)
• Use of sleep medication (a.u.)	0.0 (0.0 – 1.0)
• Daytime dysfunction (a.u.)	1.0 (1.0 – 1.0)
**Pain duration:**	
•>2 years – *n* (%)	33 (53%)
• 1–2 years – *n* (%)	8 (13%)
• 6–12 months – *n* (%)	8 (13%)
• 3–6 months – *n* (%)	13 (21%)
Pain intensity (a.u.)	5.0 (3.1 – 5.3)
Affective pain interference (a.u.)	4.5 (3.3 – 6.0)
Activity pain interference (a.u.)	5.1 (3.7 – 6.7)
Pain extension (pixels)	36120 (16069–69184)
PSD scale score:	12.5 (8.0–16.0)
• Widespread pain index score	6.0 (5.0–8.0)
• Symptom severity scale score	6.0 (3.3–8.0)

IPAQ, International Physical Activity Questionnaire; PSD, polysymptomatic distress; PSQI, Pittsburgh Sleep Quality Index; VISA-G, Victorian Institute of Sports Assessment for Gluteal Tendinopathy.

Analysis of the PSQI questionnaires showed that the median (1st – 3rd quartile) value of the global PSQI score was 9.0 (6.0–12.0), while the median (1st – 3rd quartile) sleep duration was 6.0 (5.0–7.0) hours: 50 of 62 patients were poor sleepers and 44 of 62 patients were short sleepers (42 of 62 patients were both poor and short sleepers). The sleep components that had a larger proportion of higher scores (i.e., score = 3) were sleep duration (15 of 62 patients: 24%) and sleep medication use (13 of 62 patients: 21%): the score 3 for these two components corresponds to sleep duration <5 h and use of medication three or more times a week.

Pain duration exceeded 1 year in the majority of patients (41 of 62 patients), pain intensity and activity interference were between mild and moderate in most of the cases, whereas pain affective interference was predominantly rated as moderate to severe.

The pain intensity time course showed a circadian rhythmicity over the 7-day period: as shown in Figure 1A, median values were higher in all days but one during evening (23:00) compared to both morning (8:00) and afternoon (16:00) values. Consistently, Friedman’s ANOVA showed a significant difference (*F* = 54.7, *P* < 0.0001) between the three time points (Figure 1B: data for the 7 consecutive days were pooled): pain intensity was significantly higher during evening (median: 5, 1st–3rd quartile: 3–7) compared to both morning (median: 4, 1st–3rd quartile: 3–6) and afternoon (median: 4, 1st–3rd quartile: 3–6).

**FIGURE 2:**
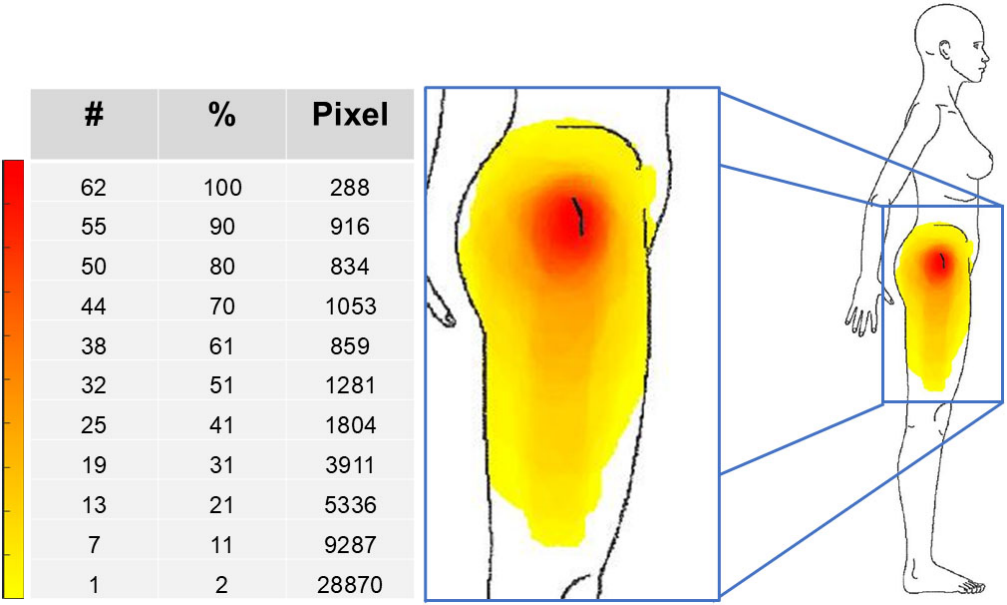
Pain frequency map for the whole group of 62 patients. The color bar represents the frequency of colored areas: red and yellow represent, respectively, the most and less frequently reported areas of pain. #, number of patients; %, percentage of patients; Pixel, number of pixels.

Pain had a peri-trochanteric distribution in all patients (median extension of 36120 pixels: red and orange areas in Figure 2), with extension to the distal portions of the lateral thigh in a few patients only. Analysis of the PSD scales showed that the median values of the WPI and SSS scores were both 6 (these scores resulted in a median PSD score of 12.5) and that 31 of 62 patients showed generalized pain (i.e., pain present in at least 4 of 5 regions). Twenty-four of the 31 patients with generalized pain had WPI score ≥7 and SSS score ≥5 (or WPI score ≥4 and SSS score ≥9, respectively). Briefly, the localized (peri-trochanteric) pain was associated with widespread pain and polysymptomatic distress symptoms in more than one-third of the patients who satisfied the fibromyalgia diagnostic criteria.

**FIGURE 3:**
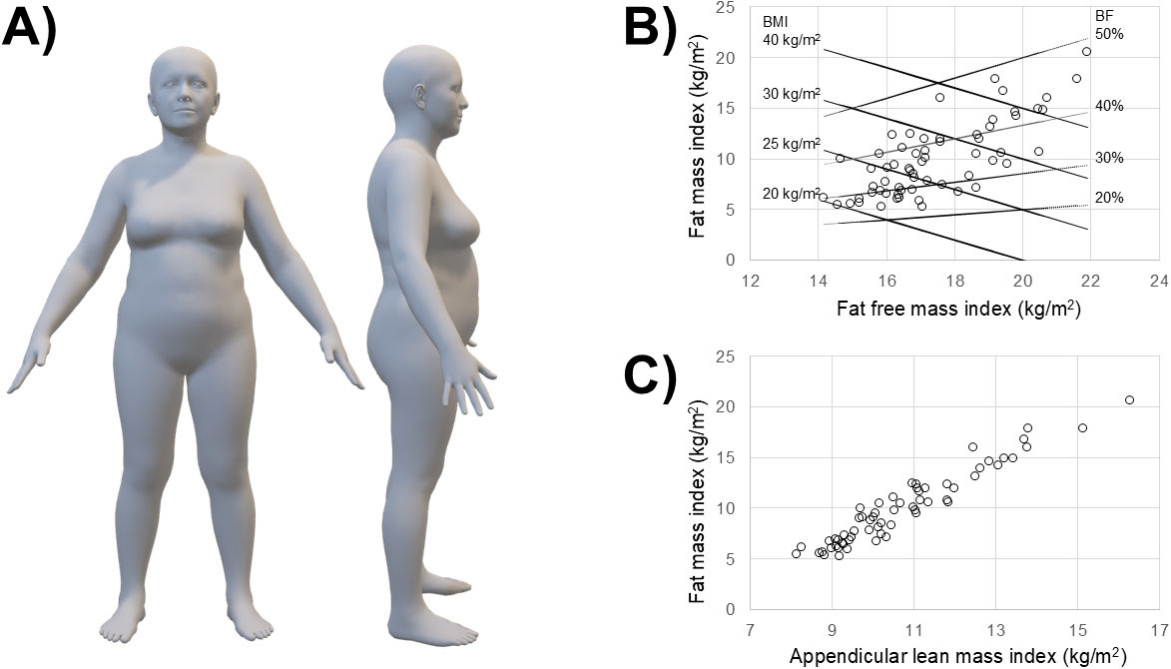
Average body shape of the 62 patients **(A)** and body composition charts showing individual values **(B,C)**: lines representing body mass index (BMI: continuous black lines) and body fat percentage (BF%: dotted black lines) are included in panel **(B)**.

The average avatar (i.e., mean body shape) of the 62 patients is shown in Figure 3A and their anthropometric and body composition characteristics are reported in Table 2. Body composition charts (Figures 3B, C) show that the higher the fat mass index, the higher both the fat free mass index and the appendicular lean mass index: 15 of 62 patients had a body mass index ≥30 kg/m^2^, 44 patients had body fat >30%, 33 patients had fat mass index >9 kg/m^2^, while none of the patients had appendicular lean mass index <5.45 kg/m^2^.

**TABLE 2:** Median (1st–3rd quartile) values of the results of the body size and composition assessments obtained in the whole group of 62 patients.

Variable	Median	1st–3rd quartile
Body mass index (kg/m^2^)	26.0	23.3 – 29.8
Waist circumference (cm)	92.7	86.0 – 102.6
Body roundness index (a.u.)	5.4	4.3 – 7.4
Hip circumference (cm)	104.5	98.9 – 112.8
Waist-to-hip ratio	0.90	0.83 – 0.92
Body fat (%)	35.2	29.6 – 40.5
Fat mass index (kg/m^2^)	9.4	6.8 – 11.9
Fat free mas index (kg/m^2^)	17.0	16.2 – 18.7
Appendicular lean mass index (kg/m^2^)	10.3	9.4 – 11.7

The waist circumference was ≥88 cm in 36 patients (the same 33 patients with increased fat mass index and 3 other patients with normal fat mass index), the body roundness index was >5 in 35 patients (30 of the 33 patients with increased fat mass index and 5 other patients with normal fat mass index), while the waist-to-hip ratio was >0.85 in 45 patients.

K-means cluster analysis of the VISA-G scores identified two subgroups of patients referred to herein as low (38 patients: median VISA-G score of 66.5%) and high (24 patients: median VISA-G score of 45.0%) severity of tendinopathy-related disability (Table 3). The two subgroups were comparable for age, pain intensity, and pain extension. The PSQI global score and the proportion of patients with poor sleep quality (PSQI score > 5) were significantly higher in the high severity subgroup compared to the low severity subgroup, while the sleep duration and the proportion of short sleepers were comparable between the two subgroups. Significant subgroup differences were observed for the PSD score as well as for the IPAQ score and physical performance: the high severity subgroup of patients showed higher PSD score and lower IPAQ score and physical performance compared to the low severity subgroup of patients. The high severity subgroup showed also higher body mass index, waist circumference, body roundness index, hip circumference, and fat mass index compared to the low severity subgroup, while no differences between the two subgroups were observed for the other body composition variables (body fat percentage, fat free mass index, and appendicular lean mass index).

**TABLE 3:** Median (1st–3rd quartile) values of the results of the clinimetric, pain phenotype, anthropometric, body composition, and physical performance assessments obtained in the two subgroups of patients with low (38 patients) and high (24 patients) severity of tendinopathy-related disability.

Variable	Low severity (*n* = 38)	High severity (*n* = 24)	*P*-value
Age (years)	59.0 (51.5–67.7)	58.0 (50.5–70.0)	0.80
Pain intensity (a.u)	4.4 (3.0–5.2)	5.1 (4.2–6.1)	0.08
Pain extension (pixels)	34574 (16670–63177)	36752 (15596–88572)	0.70
**Sleep:**			
• PSQI global score	8.0 (5.0–12.0)	9.0 (8.8–13.0)	**0.04**
• Poor sleepers (PSQI > 5)	27 of 38	23 of 24	**0.02**
• Sleep duration (hours)	6.0 (5.0–7.0)	5.6 (5.0–6.5)	0.63
• Short sleepers (duration < 7 h)	25 of 38	19 of 24	0.39
PSD scale score	11.0 (7.0–13.7)	13.0 (9.0–15.2)	**0.04**
• Widespread pain index score	6.0 (5.0–8.0)	7.5 (4.7–11.0)	0.39
• Symptom severity scale score	5.0 (3.0–7.7)	7.0 (5.0–8.2)	0.07
5-rep sit-to-stand test (s)	10.9 (9.9–12.3)	12.9 (11.4–15.4)	**0.004**
IPAQ score (MET*min/week)	2073.7 (837.7–3131.0)	462.0 (198.0–1265.5)	**0.001**
Body mass index (kg/m^2^)	25.0 (22.8–29.1)	27.7 (25.6–31.3)	**0.04**
Waist circumference (cm)	90.1 (84.6–100.9)	97.9 (92.0–108.4)	**0.04**
Body roundness index (a.u.)	4.8 (4.0–6.9)	6.1 (4.9–7.5)	**0.05**
Hip circumference (cm)	101.1 (97.8–111.0)	108.7 (103.2–116.4)	**0.03**
Waist-to-hip ratio	0.90 (0.81–0.91)	0.90 (0.89–0.94)	0.16
Body fat%	32.5 (28.5–39.6)	38.4 (33.6–40.8)	0.06
Fat mass index (kg/m^2^)	8.2 (6.7–10.6)	10.7 (8.7–12.5)	**0.05**
Fat free mass index (kg/m^2^)	16.6 (16.0–18.6)	17.1 (16.7–19.1)	0.09
Appendicular lean mass index (kg/m^2^)	10.1 (9.3–11.1)	11.1 (9.9–12.0)	0.08

Statistically significant differences are highlighted in bold. PSD, polysymptomatic distress; PSQI, Pittsburgh Sleep Quality Index; IPAQ, International Physical Activity Questionnaire.

## 4 Discussion

This is the first study investigating in patients with greater trochanteric pain syndrome the circadian rhythmicity of pain intensity and the sleep characteristics in combination with body size and composition assessed through an innovative digital anthropometric approach. The main results of this study can be summarized as follows: (i) the time course of pain intensity showed a circadian rhythmicity: pain intensity was significantly higher during evening compared to both morning and afternoon; (ii) a relevant impairment of the sleep quality and quantity was observed in most of the patients; (iii) pain had a peri-trochanteric distribution in all patients: the localized pain was also associated with widespread pain and polysymptomatic distress symptoms in more than one-third of the patients who satisfied the fibromyalgia diagnostic criteria; (iv) an increased (central) adiposity was observed in most of the patients, while the appendicular lean mass was normal; (v) subgroups of patients with different severity of tendinopathy-related disability showed differences in sleep and body composition characteristics.

Pain can be characterized along a variety of dimensions, including one of the most important distinctions: nociceptive versus neuropathic pain. Nociceptive pain results from activity in neural pathways secondary to actual or potential tissue-damaging stimuli of nociceptors by physical or chemical agents, while neuropathic pain is caused by a lesion or disease of the somatosensory nervous system. Arthritic disorders are a common example of inflammatory condition associated with nociceptive pain presenting with worse symptoms in the morning than at night, with a close temporal coupling with the circadian rhythm of the immune system activity and production of proinflammatory cytokines (41). Conversely, common conditions that are associated with neuropathic pain, such as diabetic peripheral neuropathy and postherpetic neuralgia, showed a circadian rhythmicity in symptom intensity with worse symptoms in the evening compared to morning (41, 42). To our knowledge, our study is the first investigating the circadian rhythmicity of pain intensity in patients with greater trochanteric pain syndrome and showing that the pain intensity was higher during evening compared to both morning and afternoon. Possible mechanisms underlying the observed diurnal pattern include circadian fluctuations in both extrinsic and intrinsic factors. Consistently, the mechanical stimuli over the greater trochanter may increase during evening and night because of poor posture (throughout the day and evening hours) or side sleeping (during the night), that is the position preferred by most adults as it is generally protective against spinal symptoms (43). Moreover, previous studies showed circadian fluctuations in neuroendocrine modulation of pain circuits: in fact, the levels of endogenous opioids such as beta-endorphins show a circadian rhythm with a close temporal coupling to the activity of the hypothalamic–pituitary–adrenal axis that is low during evening and night and peaks in the morning to underlie the awakening cortisol response (44–46). The observed diurnal pattern of pain intensity has relevant clinical implications for pain pharmacotherapy: patients with greater trochanteric pain syndrome might benefit more from evening intake of medications acting on the peripheral and central nervous system such as neuropathic pain medications (that can be effective also for the management of widespread pain in patients presenting a co-occurrence of tendinopathy and fibromyalgia), rather than from diurnal intake of standard pain-relief drugs such as non-steroidal anti-inflammatory drugs (that are useful to manage the nociceptive pain because they reduce inflammation, which has probably a minor pathophysiological role in tendon pain) (47).

The proper management of pain could also impact on the sleep quality and quantity. Although the link between sleep and pain is well documented (14–16) and although tendinopathic patients frequently report that the trochanteric pain may interfere with sleep (3, 17), to our knowledge our study is the first to document in patients with greater trochanteric pain syndrome a relevant impairment in sleep quality and quantity. Interestingly, the link between pain and impaired sleep is bidirectional: in fact, not only the evening pain may impact on sleep, but also restricted or poor sleep (that can be related not only to pain, but also to work and/or lifestyle environments) can be associated with greater pain (14, 15). Therefore, clinical implications of these findings are twofold: a sleep assessment should be systematically included in the evaluation of tendinopathic patients and a co-analgesic pharmacologic strategy using sleeping drugs could be proposed to tendinopathic patients presenting with impaired sleep.

The proper management of pain and sleep could also impact on the health status and the metabolic profile. The demonstration that most of our patients had increased adiposity (i.e., increased body fat percentage and fat mass index) and increases in central adiposity indices (i.e., increased waist circumference and body roundness index) confirms and extends previous studies showing an increased central adiposity in tendinopathic patients (7–10). Moreover, the observation that subgroups of patients with different severity of tendinopathy-related disability showed differences in body composition represents an original observation of our study. Interestingly, the link between increased adiposity and tendinopathy as well as the link between increased adiposity and sleep impairment are both bidirectional. In fact, not only the increased adiposity and the consequent trochanteric mechanical overload may contribute to tendinopathy (6), but also the pain-related sedentary behavior can contribute to the impairment of both physical performance and metabolic health. Moreover, not only the increased adiposity can be associated with obstructive sleep apnea syndrome (that is a well-known condition impacting on the sleep-wake cycle) and unhealthy food (i.e., high consumption of fat and carbohydrate, especially in case of evening intake) seems to negatively influence the quality of sleep (48), but also sleep deprivation and poor sleep quality contribute to energy imbalance through dysregulation of appetite hormones, increased caloric intake, and reduced physical activity (49). In addition, both pain and impaired sleep can be associated with mood disorders that, in turn, are often linked to eating disorders and unhealthy diet. The bidirectional links between increased adiposity and both pain and sleep disorders underscore the importance of integrating obesity treatment strategies into the management of tendinopathic patients, especially those presenting with widespread pain and poor or short sleep.

### 4.1 Limitations

**FIGURE 4:**
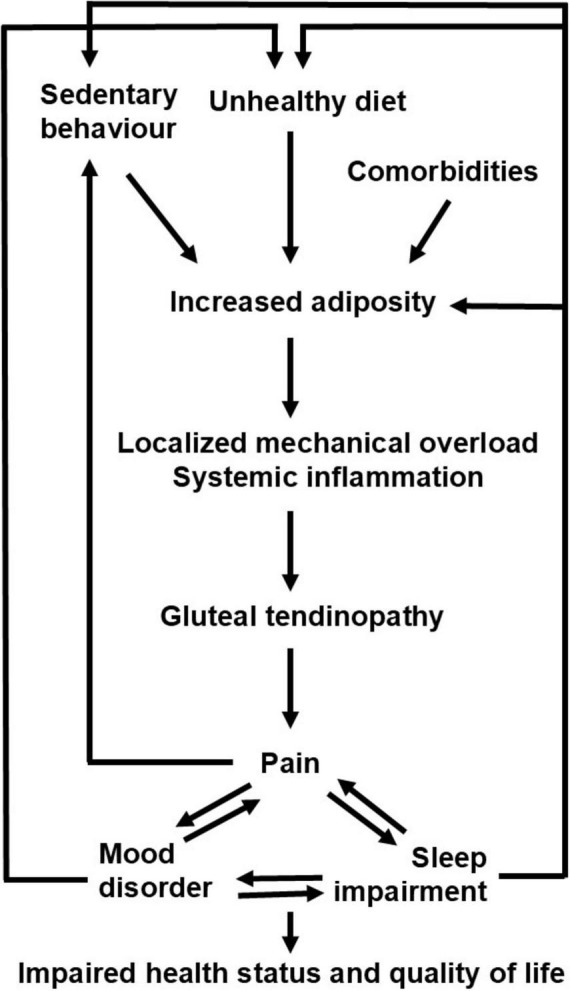
Proposed vicious cycles connecting increased adiposity, pain, and sleep impairment in patients with greater trochanteric pain syndrome.

This study has a few limitations that warrant consideration. First, the use of a convenience sample may introduce selection bias, potentially affecting the generalizability of the findings. Second, we did not investigate the possible presence of other comorbidities such as mood disorders and obstructive sleep apnea syndrome that could impact on both pain, and sleep, and physical performance. Third, we cannot infer causality given the cross-sectional design of the study: therefore, future longitudinal investigations are required to clarify whether the localized (and/or widespread) pain may contribute to the impairments in physical performance and sleep or, conversely, whether the sleep impairment may contribute to pain, increased adiposity, and impaired physical performance. However, the present and previous observations suggest that increased adiposity, pain, and impaired sleep not only feed into but exacerbate each other through distinct vicious cycles (outlined in Figure 4), driven by overlapping pathophysiological, cognitive, social mechanisms and that ultimately result in impaired health status and quality of life. We suggest that a paradigm shift in the management of patients with greater trochanteric pain syndrome is required: our data showed that the combined assessment of pain, sleep, and body composition characteristics is feasible in the clinical setting and can be useful for patient phenotyping that could be clinically relevant to propose patient-centered treatment strategies encompassing pharmacological, nutritional, and lifestyle interventions.

## 5 Conclusion

This study showed that patients with greater trochanteric pain syndrome present an impairment of sleep quality and quantity and an increased central adiposity that can be documented (and longitudinally investigated) through clinimetric and body composition assessments that should be systematically included in the evaluation of tendinopathic patients.

## Data Availability

The raw data supporting the conclusions of this article will be made available by the authors, without undue reservation.

## References

[1] Fearon A Scarvell J Neeman T Cook J Cormick W Smith P. Greater trochanteric pain syndrome: defining the clinical syndrome. *Br J Sports Med.* (2013) 47:649–53. 10.1136/bjsports-2012-091565 22983121

[2] Fearon A Cook J Scarvell J Neeman T Cormick W Smith P. Greater trochanteric pain syndrome negatively affects work, physical activity and quality of life: a case control study. *J Arthroplasty.* (2014) 29:383–6. 10.1016/j.arth.2012.10.016 24210307

[3] Fearon A. Physiotherapy management of gluteal tendinopathy. *J Physiother.* (2025) 71:81–90. 10.1016/j.jphys.2025.03.005 40175231

[4] Plinsinga M Coombes B Mellor R Nicolson P Grimaldi A Hodges P Psychological factors not strength deficits are associated with severity of gluteal tendinopathy: a cross-sectional study. *Eur J Pain.* (2018) 22:1124–33. 10.1002/ejp.1199 29427310

[5] Plinsinga M Ross M Coombes B Vicenzino B. Physical findings differ between individuals with greater trochanteric pain syndrome and healthy controls: a systematic review with meta-analysis. *Musculoskelet Sci Pract.* (2019) 43:83–90. 10.1016/j.msksp.2019.07.009 31369906

[6] Fearon A Stephens S Cook J Smith P Neeman T Cormick W The relationship of femoral neck shaft angle and adiposity to greater trochanteric pain syndrome in women. a case control morphology and anthropometric study. *Br J Sports Med.* (2012) 46:888–92. 10.1136/bjsports-2011-090744 22547561 PMC3597182

[7] Gaida J Cook J Bass S. Adiposity and tendinopathy. *Disabil Rehabil.* (2008) 30:1555–62. 10.1080/09638280701786864 18608380

[8] Gaida J Ashe M Bass S Cook J. Is adiposity an under-recognized risk factor for tendinopathy? A systematic review. *Arthritis Rheum.* (2009) 61:840–9. 10.1002/art.24518 19479698

[9] Gaida J Alfredson H Kiss Z Bass S Cook J. Asymptomatic Achilles tendon pathology is associated with a central fat distribution in men and a peripheral fat distribution in women: a cross sectional study of 298 individuals. *BMC Musculoskelet Disord.* (2010) 11:41. 10.1186/1471-2474-11-41 20196870 PMC2841085

[10] Scott A Zwerver J Grewal N de Sa A Alktebi T Granville D Lipids, adiposity and tendinopathy: is there a mechanistic link? Critical review. *Br J Sports Med.* (2015) 49:984–8. 10.1136/bjsports-2014-093989 25488953 PMC4518755

[11] Heymsfield S Bourgeois B Ng B Sommer M Li X Shepherd J. Digital anthropometry: a critical review. *Eur J Clin Nutr.* (2018) 72:680–7. 10.1038/s41430-018-0145-7 29748657 PMC6411053

[12] Minetto M Pietrobelli A Busso C Bennett J Ferraris A Shepherd J Digital anthropometry for body circumference measurements: european phenotypic variations throughout the decades. *J Pers Med.* (2022) 12:906. 10.3390/jpm12060906 35743690 PMC9224732

[13] Ahima R Lazar M. Physiology. The health risk of obesity–better metrics imperative. *Science.* (2013) 341:856–8. 10.1126/science.1241244 23970691

[14] Miettinen T Mäntyselk P Hagelberg N Mustola S Kalso E Lötsch J. Machine learning suggests sleep as a core factor in chronic pain. *Pain.* (2021) 162:109–23. 10.1097/j.pain.0000000000002002 32694382

[15] Whale K Gooberman-Hill R. The importance of sleep for people with chronic pain: current insights and evidence. *JBMR Plus.* (2022) 6:e10658. 10.1002/jbm4.10658 35866153 PMC9289983

[16] Miettinen T Sverloff J Lappalainen OP Linton SJ Sipil K Kalso E. Sleep problems in pain patients entering tertiary pain care: the role of pain-related anxiety, medication use, self-reported diseases, and sleep disorders. *Pain.* (2022) 163:e812–20. 10.1097/j.pain.0000000000002497 34561395 PMC9199106

[17] Stephens G O’Neill S Mottershead C Hawthorn C Yeowell G Littlewood C. “It’s just like a needle going into my hip, basically all of the time”. The experiences and perceptions of patients with greater trochanteric pain syndrome in the UK national health service. *Musculoskelet Sci Pract.* (2020) 47:102175. 10.1016/j.msksp.2020.102175 32452392

[18] Kong A Van der Vliet A Zadow S. MRI and US of gluteal tendinopathy in greater trochanteric pain syndrome. *Eur Radiol.* (2007) 17:1772–83. 10.1007/s00330-006-0485-x 17149624

[19] Fearon AM Ganderton C Scarvell JM Smith PN Neeman T Nash C Development and validation of a VISA tendinopathy questionnaire for greater trochanteric pain syndrome, the VISA-G. *Man Ther.* (2015) 20:805–13. 10.1016/j.math.2015.03.009 25870117

[20] Rio E Mc Auliffe S Kuipers I Girdwood M Alfredson H Bahr R ICON PART-T 2019-international scientific tendinopathy symposium consensus: recommended standards for reporting participant characteristics in tendinopathy research (PART-T). *Br J Sports Med.* (2020) 54:627–30. 10.1136/bjsports-2019-100957 31519545

[21] Fearon A Grimaldi A Mellor R Nasser A Fitzpatrick J Ladurner A ICON 2020-international scientific tendinopathy symposium consensus: the development of a core outcome set for gluteal tendinopathy. *Br J Sports Med.* (2024) 58:245–54. 10.1136/bjsports-2023-107150 38216320

[22] Caraceni A Mendoza T Mencaglia E Baratella C Edwards K Forjaz M A validation study of an Italian version of the brief pain inventory (Breve Questionario per la Valutazione del Dolore). *Pain.* (1996) 65:87–92. 10.1016/0304-3959(95)00156-5 8826494

[23] Barbero M Moresi F Leoni D Gatti R Egloff M Falla D. Test-retest reliability of pain extent and pain location using a novel method for pain drawing analysis. *Eur J Pain.* (2015) 19:1129–38. 10.1002/ejp.636 25565607

[24] Minetto MA Busso C Giannini A Meiburger K Massazza G Maffulli N. Cross-cultural adaptation and validation of the Victorian institute of sports assessment for gluteal tendinopathy questionnaire in Italian and investigation of the association between tendinopathy-related disability and pain. *Eur J Phys Rehabil Med.* (2020) 56:764–70. 10.23736/S1973-9087.20.06209-7 32638573

[25] Wolfe F Clauw D Fitzcharles M Goldenberg D Häuser W Katz R 2016 Revisions to the 2010/2011 fibromyalgia diagnostic criteria. *Semin Arthritis Rheum.* (2016) 46:319–29. 10.1016/j.semarthrit.2016.08.012 27916278

[26] Curcio G Tempesta D Scarlata S Marzano C Moroni F Rossini P Validity of the Italian version of the Pittsburgh sleep quality index (PSQI). *Neurol Sci.* (2013) 34:511–9. 10.1007/s10072-012-1085-y 22526760

[27] Buysse D Reynolds C Monk T Berman S Kupfer D. The pittsburgh sleep quality index: a new instrument for psychiatric practice and research. *Psychiatry Res.* (1989) 28:193–213. 10.1016/0165-1781(89)90047-4 2748771

[28] Minetto M Motta G Gorji N Lucini D Biolo G Pigozzi F Reproducibility and validity of the Italian version of the International physical activity questionnaire in obese and diabetic patients. *J Endocrinol Invest.* (2018) 41:343–9. 10.1007/s40618-017-0746-3 28825210

[29] Craig C Marshall A Sjöström M Bauman A Booth M Ainsworth B International physical activity questionnaire: 12-country reliability and validity. *Med Sci Sports Exerc.* (2003) 35:1381–95. 10.1249/01.MSS.0000078924.61453.FB 12900694

[30] Nasser A Fearon A Grimaldi A Vicenzino B Mellor R Spencer T Outcome measures in the management of gluteal tendinopathy: a systematic review of their measurement properties. *Br J Sports Med.* (2022) 56:877–87. 10.1136/bjsports-2021-104548 35396205

[31] Consensus Conference Panel, Watson NF Badr MS Belenky G Bliwise DL Buxton OM Joint consensus statement of the american academy of sleep medicine and sleep research society on the recommended amount of sleep for a healthy adult: methodology and discussion. *Sleep.* (2015) 38:1161–83. 10.5665/sleep.4886 26194576 PMC4507722

[32] Minetto M Busso C Ferraris A Pietrobelli A Shepherd J McCarthy C Clinical anthropometrics and body composition from 3-dimensional optical imaging. *J Vis Exp.* (2024): 10.3791/66698 38912781

[33] Loper M Mahmood N Romero J Pons-Moll G Black M. SMPL: a skinned multi-person linear model. *ACM Trans Graph*. (2015) 34:248. 10.1145/2816795.2818013

[34] Meshcapade. Available online at: https://meshcapade.com/ (accessed October 27, 2025).

[35] Ng B Sommer M Wong M Pagano I Nie Y Fan B Detailed 3-dimensional body shape features predict body composition, blood metabolites, and functional strength: the Shape Up! studies. *Am J Clin Nutr.* (2019) 110:1316–26. 10.1093/ajcn/nqz218 31553429 PMC6885475

[36] Thomas D Bredlau C Bosy-Westphal A Mueller M Shen W Gallagher D Relationships between body roundness with body fat and visceral adipose tissue emerging from a new geometrical model. *Obesity.* (2013) 21:2264–71. 10.1002/oby.20408 23519954 PMC3692604

[37] Ross R Neeland I Yamashita S Shai I Seidell J Magni P Waist circumference as a vital sign in clinical practice: a consensus statement from the IAS and ICCR Working group on visceral obesity. *Nat Rev Endocrinol.* (2020) 16:177–89. 10.1038/s41574-019-0310-7 32020062 PMC7027970

[38] Kelly T Wilson K Heymsfield S. Dual energy X-Ray absorptiometry body composition reference values from NHANES. *PLoS One.* (2009) 4:e7038. 10.1371/journal.pone.0007038 19753111 PMC2737140

[39] Baumgartner R Koehler K Gallagher D Romero L Heymsfield S Ross R Epidemiology of sarcopenia among the elderly in New Mexico. *Am J Epidemiol.* (1998) 147:755–63. 10.1093/oxfordjournals.aje.a009520 9554417

[40] Klukowska A Staartjes V Vandertop W Schröder M. Five-repetition sit-to-stand test performance in healthy individuals: reference values and predictors from 2 prospective cohorts. *Neurospine.* (2021) 18:760–9. 10.14245/ns.2142750.375 35000330 PMC8752709

[41] Segal J Tresidder K Bhatt C Gilron I Ghasemlou N. Circadian control of pain and neuroinflammation. *J Neurosci Res.* (2018) 96:1002–20. 10.1002/jnr.24150 28865126

[42] Gilron I Bailey J Vandenkerkhof E. Chronobiological characteristics of neuropathic pain: clinical predictors of diurnal pain rhythmicity. *Clin J Pain.* (2013) 29:755–9. 10.1097/AJP.0b013e318275f287 23370066

[43] Cary D Briffa K McKenna L. Identifying relationships between sleep posture and non-specific spinal symptoms in adults: a scoping review. *BMJ Open.* (2019) 9:e027633. 10.1136/bmjopen-2018-027633 31256029 PMC6609073

[44] Petraglia F Facchinetti F Parrini D Micieli G De Luca S Genazzani A. Simultaneous circadian variations of plasma ACTH, beta-lipotropin, beta-endorphin and cortisol. *Horm Res.* (1983) 17:147–52. 10.1159/000179690 6303934

[45] Iranmanesh A Lizarralde G Johnson M Veldhuis J. Circadian, ultradian, and episodic release of beta-endorphin in men, and its temporal coupling with cortisol. *J Clin Endocrinol Metab.* (1989) 68:1019–26. 10.1210/jcem-68-6-1019 2524500

[46] Minetto M Lanfranco F Tibaudi A Baldi M Termine A Ghigo E. Changes in awakening cortisol response and midnight salivary cortisol are sensitive markers of strenuous training-induced fatigue. *J Endocrinol Invest.* (2008) 31:16–24. 10.1007/BF03345561 18296900

[47] Jomaa G Kwan C Fu S Ling S Chan K Yung P A systematic review of inflammatory cells and markers in human tendinopathy. *BMC Musculoskelet Disord.* (2020) 21:78. 10.1186/s12891-020-3094-y 32028937 PMC7006114

[48] Muscogiuri G Barrea L Annunziata G Di Somma C Laudisio D Colao A Obesity and sleep disturbance: the chicken or the egg? *Crit Rev Food Sci Nutr.* (2019) 59:2158–65. 10.1080/10408398.2018.1506979 30335476

[49] Figorilli M Velluzzi F Redolfi S. Obesity and sleep disorders: a bidirectional relationship. *Nutr Metab Cardiovasc Dis.* (2025) 35:104014. 10.1016/j.numecd.2025.104014 40180826

