# Supplementary material for: Increased adiposity and impaired sleep are associated with severity of greater trochanteric pain syndrome: a cross-sectional study
Source: Front Med (Lausanne). 2025 Dec 3;12:1718267. doi: 10.3389/fmed.2025.1718267 (PMC12708603; doi:10.3389/fmed.2025.1718267)
Supplement: Supplementary file 1 [file Data_Sheet_1.pdf]

VARIABLE	*EQUATION*	*REF*
Arm lean (kg) – female subject	*3.83 + 0.05 x weight – 0.04 x (hips at width max circumference) – 1.35 x waist-to-hip ratio*	***1***
Leg lean (kg) – female subject	*5.81 – 0.02 x age + 0.13 x weight – 0.03 x (hips at width max circumference) – 7.45 x waist-to-height ratio*	***1***
Body roundness index	$364.2 - 365.5 \times \{1 - [(0.5 \times \text{waist circumference} / \pi)^2 / (0.5 \times \text{height})^2]\}^{0.5}$	***2***

Supplementary table 1.
Prediction equations for the body fat percentage and appendicular lean mass.
Circ.= circumference.
Units of measurement for lean equations: years for age, lbs for weight, inch. for circumference
Units of measurement for body roundness index equation: waist circumference and height are expressed in meters

1. Ng BK, Sommer MJ, Wong MC, Pagano I, Nie Y, Fan B, et al. Detailed 3-dimensional body shape features predict body composition, blood metabolites, and functional strength: the Shape Up! studies. *Am J Clin Nutr.* (2019) 110:1316-26. doi: 10.1093/ajcn/nqz218

2. Thomas DM, Bredlau C, Bosy-Westphal A, Mueller M, Shen W, Gallagher D, et al. Relationships between body roundness with body fat and visceral adipose tissue emerging from a new geometrical model. *Obesity (Silver Spring).* (2013) 21:2264-71. doi: 10.1002/oby.20408
